# Association between dietary vitamin C and telomere length: A cross-sectional study

**DOI:** 10.3389/fnut.2023.1025936

**Published:** 2023-01-26

**Authors:** Yuan Cai, Yu-di Zhong, Hao Zhang, Pei-lin Lu, Yong-yi Liang, Biao Hu, Hui Wu

**Affiliations:** ^1^Department of Radiology, The Second Affiliated Hospital of Guangzhou Medical University, Guangzhou, China; ^2^Guangzhou Medical University, Guangzhou, China; ^3^Guangdong Ocean University, Zhanjiang, China; ^4^Department of Medical Imaging, The Second Clinical School of Guangzhou Medical University, Guangzhou, China; ^5^Department of Clinical Medicine, Guangzhou Medical University, Guangzhou, China

**Keywords:** telomere length, vitamin C, interaction, cross-sectional studies, National Health and Nutrition Examination Surveys

## Abstract

**Background:**

Currently, telomere length is known to reflect the replication potential and longevity of cells, and many studies have reported that telomere length is associated with age-related diseases and biological aging. Studies have also shown that vitamin C acts as an oxidant and free radical scavenger to protect cells from oxidative stress and telomere wear, thus achieving anti-aging effects. At present, there are few and incomplete studies on the relationship between vitamin C and telomere length, so this study aims to explore the relationship between vitamin C and telomere length.

**Methods:**

This study used cross-sectional data from the National Health and Nutrition Examination Surveys (NHANES) database from 1999 to 2002, a total of 7,094 participants were selected from all races in the United States. Male participants accounted for 48.2% and female participants accounted for 51.8%. The correlation between vitamin C and telomere length was assessed using a multiple linear regression model, and the effect of dietary vitamin C on telomere length was obtained after adjusting for confounding factors such as age, gender, race, body mass index (BMI), and poverty income ratio (PIR).

**Results:**

This cross-sectional study showed that vitamin C was positively correlated with telomere length, with greater dietary vitamin C intake associated with longer telomeres (β = 0.03, 95% CI: 0.01–0.05, *P* = 0.003).

**Conclusion:**

This study shows that vitamin C intake is positively correlated with human telomere length, which is of guiding significance for our clinical guidance on people’s health care, but our study need to be confirmed by more in-depth and comprehensive other research results.

## Introduction

Telomere is a DNA-protein complex composed of TTAGGG-DNA repeats and a small amount of protective binding proteins located at the end of chromosomes. Telomere will shorten with cell division or attack. When the telomere is shortened to a certain extent, the cell will no longer divide, thus affecting the complement of organs and tissues to cells (1). Studies have shown that there is a causal relationship between telomere length and cellular senescence and body senescence, and telomere length can be considered as a marker of cellular aging (2). In addition, telomere length is also believed to be associated with the incidence, progression, and mortality of several age-related diseases, including cardiovascular and cerebrovascular diseases, Alzheimer’s disease (3), cancer (4), etc (5).

Vitamin C (ascorbic acid) is an essential dietary nutrient for human body and has a variety of biological functions. Studies have shown that vitamin C, with its special chemical structure and properties, can produce multiple positive responses to the immune system, endothelial integrity, and lipoprotein synthesis by mediating different pathways, such as the oxidative pathway and the mitochondrial pathway (6). Its electron-providing ability makes it a potent antioxidant and free radical scavenger (7, 8). The body cannot synthesize these nutrients on its own, so proper intake of vitamin C is important for maintaining good health (9).

Telomeres shorten when attacked by free radicals. Studies have shown that telomere associations are the result of decreased telomerase activity and that increased reactive oxygen species (ROS) levels are accompanied by defective telomerase activity (10). Telomere is attacked by free radicals and shortened. Vitamin C is a free radical scavenger in the body, and telomere length is a sign of cell aging. As an antioxidant, vitamin C is often used as one of the formulas for skin care products such as anti-aging and whitening (11). All of these studies and applications (1, 11) seem to confirm a potential link between vitamin C and telomere length, but the mechanism of action is still unclear. Some previous studies have investigated the association between blood vitamin C concentrations and telomere length (12), as well as the effect of vitamin use on telomere length in women (13), but these studies had small sample sizes and did not fully investigate the possible influencing factors of the association between dietary vitamin C intake and telomere length. Therefore, this study will use data from National Health and Nutrition Examination Surveys (NHANES) to perform statistical studies to complement current research on the association between dietary vitamin C intake and telomere length.

## Materials and methods

### Study design and participants

The Centers for Disease Control and Prevention has conducted the NHANES since 1960. The program is approved by the National Center for Health Statistics (NCHS) Research Ethics Review Board and operated by the NCHS. Health statistics on about 5,000 people in different states are collected each year in an effort to understand the health and nutrition of adults and children in the United States. Written informed consent was obtained from all study participants.

Data from 1999 to 2000 and 2001 to 2002 were combined for analysis. Among the 21,004 participants, 13,177 participants with unknown leukocyte telomere length (LTL) data and 348 participants with unknown vitamin C data were excluded, and 385 participants with smoke stoke and marry unknown, we finally obtained a sample of 7,094 participants.

### Telomere length assessment

Telomere length was measured in the laboratory of Dr. Elizabeth Blackburn (14) at the University of California. The strategy for measuring relative LTL using quantitative polymerase chain reaction (PCR) is to measure the ratio of the number of duplicate copies of the telomere for each DNA sample and reference sample to the number of copies of the single copy gene, which is proportional to the average telomere length. The number of telomere repeats in each sample was measured as the dilution level of the reference DNA sample, which is the number of reaction cycles required for the PCR product of a single copy gene. That is, the dilution factor ratio is the ratio of relative telomere to single copy gene (T/S). The ratio of telomere length to standard reference DNA (T/S) was obtained for participants aged 20 years and older. Each sample was measured on repeated wells, three times on different days. The number of runs (<6% runs) of plates with more than eight invalid control wells and more than four control DNA values were excluded. After excluding potential outliers for each sample, the mean and standard deviation of the T/S ratio were calculated, and the coefficient of variation between measurements was 6.5%. According to the formula [3,274 + 2,413* (T/S)], DNA samples from human diploid fibroblast cell line IMR90 were analyzed and compared with the telomere fragment length by Southern blot analysis, and T/S ratio and base pair (bp) conversion were calculated.

### Dietary vitamin C intake

The dietary data came from a comprehensive interview called What We Eat in America (WWEIA). Dietary intake data were obtained by the 24-h dietary interview method, in which respondents recalled the types and quantities of food and beverages consumed in the past 24 h. Data were collected through the computer-assisted dietary interview system, and food intake was evaluated for energy, nutrients, and other components. Dietary intake of vitamin C was estimated from the United States Department of Agriculture (USDA) Survey Nutrient Database.

### Statistical analysis

The statistical package R^1^ (R Foundation) was used for data analysis in this study. We used means and standard deviations or medians and quartiles to describe continuous variables, which were assessed by chi-square tests and *t*-tests; weighted percentages were used to describe categorical variables. Calculate 95% confidence intervals. The level of statistical significance was set at *P* < 0.05. Study using multiple linear regression analysis and three adjustment model is established to evaluate the correlation between dietary vitamin C and telomere. Model 1 adjusted for gender, age, and race, model 2 adjusted for including model 1 and physical activity, body mass index (BMI), and poverty income ratio (PIR), and model 3 further adjusted for diabetes, hypertension, smoking habits, alcohol intake, stroke, and cardiovascular disease. The results are described using coefficients β and corresponding 95% confidence intervals.

### Covariates

Based on previous studies of telomere length, the following covariates will be added to construct the adjustment model. The continuous variables include age and the poverty to income ratio (PIR). Categorical variables included race (Mexican American, non-Hispanic black, non-Hispanic white, other Hispanic, other race–including multiracial), marital status (married, unmarried), BMI (25, 25–29.9, ≥30 kg/m^2^), smoking status (never = smoked <100 cigarettes in life, former = smoked <100 cigarettes in life and smoke not at all now, now = smoked moth than 100 cigarettes in life, and smoke some days or every day), Alcohol consumption (yes = at least 12 alcohol drinks per year vs. no = < 12 alcohol drinks per year), CVD is determined by any reported diagnosis of congestive heart failure, coronary heart disease, angina, heart attack, or stroke. Residents were asked “Has a doctor or other health professional ever told you that you have congestive heart failure/coronary heart disease/angina/heart attack/stroke?” and participants who answered “yes” to either question were included in our study’s general cardiovascular disease group. A sample will be considered as hypertensive if it is diagnosed by a physician and treated with hypertension medication or measured blood pressure ≥ 140/90 mmHg, ambulatory blood pressure monitoring: mean blood pressure ≥ 130/80 mmHg within 24 h, daytime ≥ 135/85 mmHg, at night ≥ 130/80 mmHg. Participants were considered diabetic when they met any of the following criteria:(1) had been diagnosed with all types of diabetes; (2) fasting blood glucose >7.0 mmol/L; (3) random blood glucose or 2-h The oral glucose tolerance test (OGTT) blood glucose >11.1 mmol/L; (4) had used diabetes medications or insulin.

## Results

### Baseline characteristics of study

#### Population

**FIGURE 1:**
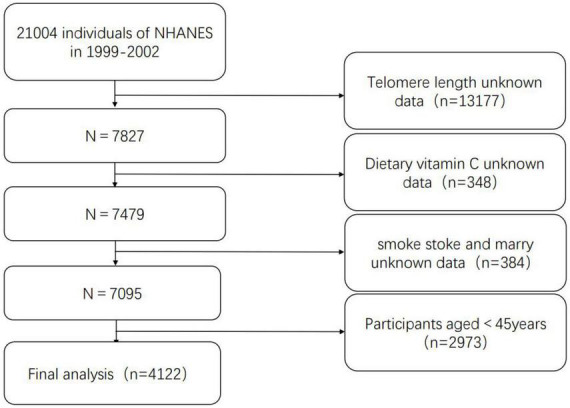
Flow chart of the study participants.

The data of 1999–2000 and 2001–2002 from NHANES database were used in this study. A total of 7,094 participants were finally included in the analysis after excluding those with unknown telomere length data, unknown dietary vitamin C intake, unknown marry, and smoke stoke. The exclusion criteria and results are shown in Figure 1. According to the quantile of dietary vitamin C intake, the characteristics are described in Table 1. Compared with Tertile 1 (≤41.5) and Tertile 2 (41.5–107.75), and Tertile 3 (≥107.75) participants were more male and Hispanic white, had higher PIR, consumed less alcohol, and had lower rates of smoking and diabetes.

**TABLE 1:** Baseline characteristics of study participants.

Variables	Total (*n* = 7,094)	Dietary vitamin C intake (mg/day)			*P*-value
		≤41.5 (*n* = 2,366)	41.5–107.75 (*n* = 2,363)	≥107.75 (*n* = 2,365)	
Year cycle [*n* (%)]					0.011
1999–2000	3,063 (43.2)	963 (40.7)	1,043 (44.1)	1,057 (44.7)	
2000–2001	4,031 (56.8)	1,403 (59.3)	1,320 (55.9)	1,308 (55.3)	
Age [Median (IQR)]					
Gender [*n* (%)]					0.047
Female	3,677 (51.8)	1,260 (53.3)	1,239 (52.4)	1,178 (49.8)	
Male	3,417 (48.2)	1,106 (46.7)	1,124 (47.6)	1,187 (50.2)	
Race [*n* (%)]					<0.001
Mexican American	1,695 (23.9)	535 (22.6)	545 (23.1)	615 (26)	
Non-Hispanic black	1,186 (16.7)	431 (18.2)	370 (15.7)	385 (16.3)	
Non-Hispanic white	3,628 (51.1)	1,212 (51.2)	1,249 (52.9)	1,167 (49.3)	
Other Hispanic	373 (5.3)	130 (5.5)	135 (5.7)	108 (4.6)	
Other race-including multiracial	212 (3.0)	58 (2.5)	64 (2.7)	90 (3.8)	
Marital status [*n* (%)]					0.163
No	1,048 (14.8)	375 (15.8)	329 (13.9)	344 (14.5)	
Yes	6,046 (85.2)	1,991 (84.2)	2,034 (86.1)	2,021 (85.5)	
BMI (Mean ± SD)	28.4 ± 6.2	28.6 ± 6.4	28.4 ± 6.1	28.0 ± 6.0	0.005
Smoking status [*n* (%)]					<0.001
Never	3,661 (51.6)	1,094 (46.2)	1,226 (51.9)	1,341 (56.7)	
Former	1,919 (27.1)	591 (25)	675 (28.6)	653 (27.6)	
Now	1,514 (21.3)	681 (28.8)	462 (19.6)	371 (15.7)	
Stroke [*n* (%)]					0.329
No	6,883 (97.0)	2,286 (96.6)	2,295 (97.1)	2,302 (97.3)	
Yes	211 (3.0)	80 (3.4)	68 (2.9)	63 (2.7)	
CVD [*n* (%)]					0.251
No	6,346 (89.5)	2,112 (89.3)	2,099 (88.8)	2,135 (90.3)	
Yes	748 (10.5)	254 (10.7)	264 (11.2)	230 (9.7)	
Hypertension [*n* (%)]					0.235
No	3,982 (56.1)	1,319 (55.7)	1,303 (55.1)	1,360 (57.5)	
Yes	3,112 (43.9)	1,047 (44.3)	1,060 (44.9)	1,005 (42.5)	
DM [*n* (%)]					0.048
No	5,510 (77.7)	1,878 (79.4)	1,810 (76.6)	1,822 (77)	
Yes	1,584 (22.3)	488 (20.6)	553 (23.4)	543 (23)	
Drinks day (Mean ± SD)	2.8 ± 2.8	3.1 ± 3.0	2.7 ± 2.7	2.7 ± 2.6	<0.001
*Leukocyte telomere length* (Mean ± SD)	1.0 ± 0.3	1.0 ± 0.3	1.0 ± 0.3	1.0 ± 0.3	0.179
PIR [Median (IQR)]	2.4 (1.2, 4.3)	2.1 (1.1, 3.9)	2.5 (1.3, 4.4)	2.6 (1.3, 4.8)	<0.001

### Association of dietary vitamin C (mg/day) with leukocyte telomere length (kp)

**FIGURE 2:**
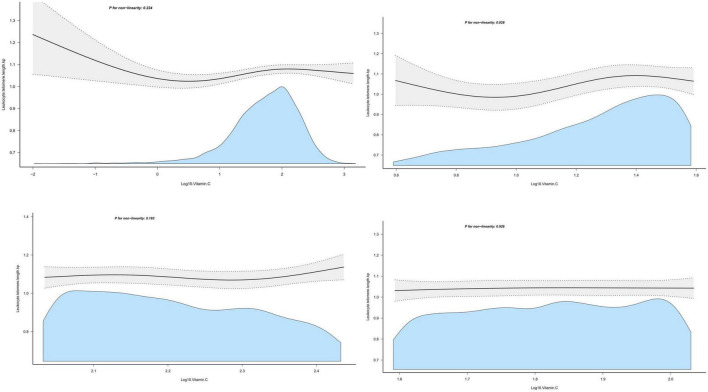
The curve fitting of leukocyte telomere length (LTL) and vitamin C.

**FIGURE 3:**
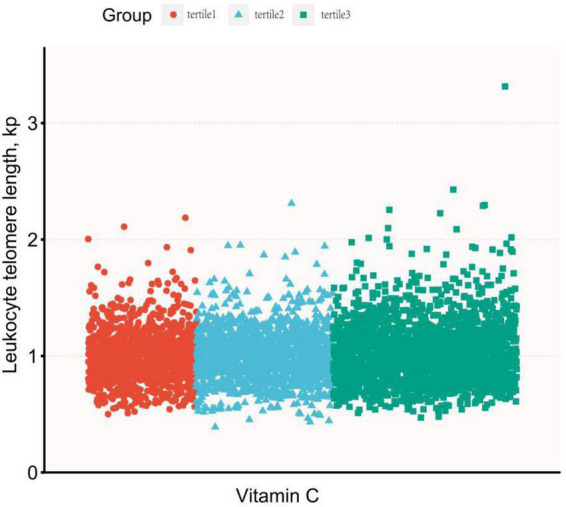
The scatter plot of leukocyte telomere length (LTL) and vitamin C.

Dietary vitamin C intake was log-transformed and described with continuous variables, and the association between dietary vitamin C intake and LTL was expressed using multiple linear regression analysis, as shown in Table 2. In the original data model, there was no statistically significant relationship between dietary vitamin C intake and LTL (β = 0.01, 95% CI: 0–0.02, *P* = 0.155), however, after adjusting for different confounders, dietary vitamin C intake was positively correlated with longer LTL, with *P* < 0.01. In the fully adjusted model (Model 3), a one-unit increase in dietary vitamin C intake after conversion was significantly associated with longer telomere length (β = 0.03, 95% CI: 00.1–0.05, *P* = 0.001). At the same time, dietary vitamin C intake was described as a categorical variable using the rule of thirds, and the results are shown in Table 3. It was still observed that participants with higher dietary vitamin C intake were associated with longer LTL (β = 0.04, 95% CI: 0.01–0.06, *P* = 0.002). *P* linear trend tests also indicated a linear relationship between LTL and the quantile of dietary vitamin C intake (Figure 2 shows the curve fitting of LTL and vitamin C. Figure 3 shows the Scatter plot of LTL and vitamin C).

**TABLE 2:** Crude model: Not adjusted.

Models	*n*	Leukocyte telomere length (kp)	
		β (95% CI)	*P*-value
Crude model	7,094	0.01 (0∼0.02)	0.051
Model 1	7,094	0.02 (0.01∼0.03)	<0.001
Model 2	7,094	0.02 (0.01∼0.03)	0.001
Model 3	7,094	0.02 (0.01∼0.03)	0.005

Model 1: Adjusted for year cycle, age, gender, and race.

Model 2: Model 1 + marital status, body mass index (BMI), and poverty income ratio (PIR).

Model 3: Model 2 + DM, hypertension, smoking status, drinking days, stroke, and cardiovascular disease (CVD).

**TABLE 3:** Crude model: Not adjusted.

Dietary vitamin C intake (mg/day)	Leukocyte telomere length (kp)							
	Crude model		Model 1		Model 2		Model 3	
	β (95% CI)	*P*-value	β (95% CI)	*P*-value	β (95% CI)	*P*-value	β (95% CI)	*P*-value
**Tertiles**								
≤41.5	0 (Ref)		0 (Ref)		0 (Ref)		0 (Ref)	
41.5–107.75	0 (−0.02∼0.01)	0.865	0.01 (0∼0.03)	0.029	0.01 (0∼0.03)	0.066	0.01 (0∼0.03)	0.108
≥107.75	0.01 (0∼0.03)	0.129	0.03 (0.01∼0.04)	<0.001	0.02 (0.01∼0.04)	0.001	0.03 (0.01∼0.05)	0.003
P for trend	0.01 (0∼0.01)	0.13	0.01 (0.01∼0.02)	<0.001	0.01 (0.01∼0.02)	0.001	0.01 (0∼0.02)	0.003

Model 1: Adjusted for year cycle, age, gender, and race.

Model 2: Model 1 + marital status, body mass index (BMI), and poverty income ratio (PIR).

Model 3: Model 2 + DM, hypertension, smoking status, drinking days, stroke, and CVD.

## Discussion

In this study, after adjusting for multiple linear regression and confounding factors, dietary vitamin C intake was positively correlated with LTL. We found that when model 2 adjusted for covariates including BMI and diabetes prevalence, the correlation significance decreased, while when model 3 added other covariates, the correlation significance increased. It suggests that vitamin C may influence telomere length through a number of potential pathways, and the following studies can explain the association found. Previous studies have shown that vitamin C activates telomerase activity, so that the shortened DNA sequence can be repaired to a certain extent. Fraga et al. (15) pointed out that ascorbate can prevent DNA damage in cells with high proliferation and differentiation ability. Telomerase, as a reverse transcriptase, can prolong telomeric DNA. It antagonizes the expression of telomere sequence deletion after DNA replication, preventing telomere sequence from being too short. In fact, telomerase is rarely expressed or not very active in most normal cells, which is not conducive to maintaining a proper range of telomere length (16). The shortening of LTL also means that the cell proliferation ability is reduced, that is, cell senescence (17). Tsoukalas et al. (18) used a natural telomere activator that contains vitamin C and found an increase in telomerase expression in the brains of mice; The study of Wei et al. (19). Also proved that vitamin C can increase human telomerase activity. Although the specific mechanism by which vitamin C can improve telomerase activity is not clear, which may be related to the increased expression of enzyme modified proteins, there is a possibility that vitamin C can prolong the shortened DNA sequence after cell division by increasing telomerase activity.

Previous studies have examined the association between multivitamin use and telomere length in women (13), as well as the effect of high concentrations of nutrients such as lutein on telomere length (12). These studies reached similar conclusions to this one, the first of which used only 586 participants from the sister study, although they were recently screened and adjusted for confounding factors. Our study still has some advantages. First, we had 4,122 participants, and the participants were not limited to women. Moreover, based on their study, we delve into the correlation between vitamin C intake and telomeres. The study by Surtees et al. (20) showed no association between vitamin C and telomeres in 4,441 female participants in a homogeneous population. The reason for the difference may be due to the fact that only women were selected for the study and the geographical location of the participants. Their study further suggests that setting a more determined oral vitamin C content can be used to investigate whether there is a dose-response relationship between vitamin C and LTL. This is more conducive to solving the problem of vitamin C supplementation and cell aging in the Chinese population. At the same time, compared with some prospective studies, such as Myers et al. (21), on the effect of maternal vitamin C intake on infant telomere length, we noted some limitations of this study. As a cross-sectional study, this study could not prove a causal relationship between dietary vitamin C and LTL. Behavioral differences, such as exercise habits, cannot be measured, so there are more confounding factors that cannot be controlled for. We also noted that in our study, the dietary data obtained came from the interviewees’ recall interviews and were then converted into intake according to the formula, which would lead to certain errors in the final dietary intake of vitamin C, because ascorbic acid, namely, vitamin C, has various molecular forms. Medical practice has proved that only L-ascorbic acid is involved in a variety of reactions in human body and has research value (11). However, our study only calculated dietary vitamin C intake based on dietary interview data, lacking qualitative and quantitative analysis of the proportion and quantity of L-ascorbic acid in vitamin C intake. This may lead to discrepancies in the data used to analyze the relationship between vitamin C and LTL, which may not reflect the true association between the two. In addition, it is possible for other molecular forms of vitamin C to have other reactions or affect the effects of L-ascorbic acid in the body.

We used data from 24-h dietary recall to analyze the effect of vitamin C on telomere length by comparing different amounts of energy, nutrients, and other components consumed by different people. However, due to the limitation of the NHANES database, we could not obtain the data of telomere length changes before and after vitamin C intake, which is also the deficiency of the subject design of our paper.

## Data availability statement

Publicly available datasets were analyzed in this study. This data can be found here: https://www.cdc.gov/nchs/nhanes.

## Ethics statement

Ethical review and approval was not required for the study on human participants in accordance with the local legislation and institutional requirements. The patients/participants provided their written informed consent to participate in this study.

## Author contributions

BH, YC, and Y-DZ: conception and design. HZ and P-LL: consulting relevant literature and writing articles. HW: administrative support. Y-YL: collection of relevant data. All authors contributed to the data analysis and interpretation, manuscript writing, and final approval of manuscript.

## Footnotes

1
http://www.R-project.org
